# Quality of Life in Ukrainian Children and Adolescents with Cancer Relocated to Switzerland During the Russian–Ukrainian War: An Exploratory Multicenter Study

**DOI:** 10.3390/curroncol33090553

**Published:** 2026-09-11

**Authors:** Rahel Kasteler, Ahmed Farrag, Andreas Klein-Franke, Calogero Mazzara, Francesco Ceppi, Cornelia Vetter, Nicolas von der Weid, Katrin Scheinemann

**Affiliations:** 1 Pediatric Hematology-Oncology Center, Children’s Hospital of Eastern Switzerland, 9000 St. Gallen, Switzerlandkatrin.scheinemann@kispisg.ch (K.S.); 2 Department of Oncology, Hematology, Immunology, Stem Cell Transplantation and Somatic Gene Therapy, University Children’s Hospital Zurich-Eleonore Foundation, 8008 Zurich, Switzerland; 3 Department of Pediatrics, Division of Pediatric Hematology and Oncology, Children’s Hospital of Central Switzerland, 6004 Lucerne, Switzerland; 4 Pediatric Oncology Department, South Egypt Cancer Institute, Assiut University, Assiut 71515, Egypt; 5 Division of Pediatric Hematology and Oncology, Cantonal Hospital Aarau, 5001 Aarau, Switzerland; 6 Department Woman-Mother-Child, Pediatric Hematology-Oncology Unit, Division of Pediatrics Lausanne University Hospital (CHUV) and University of Lausanne (UNIL), 1011 Lausanne, Switzerland; 7 Department of Pediatric Hematology and Oncology, University Children’s Hospital Basel (UKBB), University of Basel, 4031 Basel, Switzerland; 8 Faculty of Health Sciences and Medicine, University of Lucerne, 6002 Lucerne, Switzerland

**Keywords:** quality of life, PedsQL, pediatric oncology, childhood cancer, refugees, Ukraine

## Abstract

The war in Ukraine, beginning in February 2022, disrupted continuous medical care for Ukrainian childhood and adolescent cancer patients (UCC), many of whom were relocated to pediatric cancer centers worldwide, including Switzerland. In this Brief Report, we describe their health-related quality of life (HRQoL) after arrival. In a multicenter, cross-sectional survey across five Swiss pediatric oncology centers, we included patients ≤18 years at diagnosis who arrived after 24 February 2022 and were undergoing active treatment. HRQoL was assessed by the PedsQL™ 4.0 Generic Core Scales (self- and parent-reports). Fourteen of 23 eligible families (61%) participated. HRQoL declined with age, particularly in physical functioning, while psychosocial functioning remained relatively stable but lower among adolescents; parent scores closely matched self-reports. Scores were referenced against a published cohort of healthy Ukrainian children and previously reported pediatric cancer populations. Given the small sample and lack of a matched control group, the findings cannot separate the effects of displacement, cancer, and treatment and are hypothesis-generating. They suggest a potential role for age-tailored supportive care in displaced children with cancer and call for larger, controlled studies.

## Simple Summary

When war broke out in Ukraine in 2022, many families whose children were being treated for cancer had to flee abroad, interrupting essential medical care. Little is known about how these children and their families cope after arrival. We surveyed fourteen families at five Swiss children’s cancer centers to learn about the children’s well-being. We found that older children and teenagers tended to report a lower quality of life, especially physical functioning, while emotional and social well-being varied less across age. This exploratory study was small, so these preliminary findings cannot separate the effects of displacement, cancer and treatment, but the results can be used for hypothesis generation. The findings suggest that displaced children with serious illnesses need support tailored to their age, which will help clinics and policymakers plan better care in future crises.

## 1. Introduction

The war in Ukraine, which began in February 2022, has profoundly disrupted healthcare delivery for both those remaining in the country and refugees. Children and adolescents with pre-existing medical conditions, particularly cancer, represent an especially vulnerable population. A recent scoping review has confirmed that these disruptions extended across pediatric healthcare, affecting access to care and children’s mental health, and has identified the resulting evidence gaps as a research priority [1]. Early in the conflict, access to continuous oncological care was no longer assured for approximately 1000 children and adolescents with cancer in Ukraine [2,3,4]. As part of international relief efforts, 37 Ukrainian children and adolescents with cancer (UCC) and their families were relocated to Switzerland during the first year of the war, supported by the SAFER Ukraine initiative coordinated by St. Jude Children’s Research Hospital (Tennessee, USA) [3,5], additional patients arrived through private efforts.

In Switzerland, around 360 new cases of pediatric cancer are diagnosed annually [6]. The arrival of UCC therefore represented an estimated increase of nearly 10–20% in patients requiring pediatric oncological care. To manage this, the Swiss Society of Pediatric Hematology and Oncology (SSPHO) coordinated the allocation of these children and adolescents among all nine Swiss pediatric oncology centers. UCC receive the same level of oncological treatment and psychosocial support as Swiss patients.

Children and adolescents with cancer and their families experience impaired health-related quality of life (HRQoL) and require comprehensive supportive care, including psychosocial, educational, and practical resources [7,8,9]. Poku et al. describe barriers to accessing care and identifies post-migration needs as under-researched in children and young people with chronic illness in the context of migration [10]. For UCC, these needs may be impacted by displacement, war-related trauma, and barriers related to language and culture, potentially challenging continuity of care and further impacting quality of life [11].

The present study aimed to survey HRQoL among UCC living in Switzerland after the start of the Ukrainian-Russian war using validated age-specific instruments and to explore how HRQoL differed across age groups.

## 2. Methods

### 2.1. Study Design and Population

This was a multicenter, cross-sectional, questionnaire-based survey. Eligible participants included Ukrainian children and adolescents with cancer, classified according to the International Classification of Childhood Cancer, Third Edition (ICCC-3) [12]. Patients were ≤18 years at diagnosis, had arrived in Switzerland after 24 February 2022 (onset of the war in Ukraine) and before the start of recruitment, and were receiving care at one of five pediatric oncology centers in Switzerland, out of nine nationwide (Aarau, Basel, Lausanne, Lucerne, St. Gallen). Patients in palliative care settings were excluded. Ethical approval was obtained from the Ethics Committee of Eastern Switzerland (EKOS 24/008).

### 2.2. Study Procedures

Eligible UCC and their families were contacted by mail at their last known address to the respective treatment hospital. The first postal mailing included study information, informed consent forms, the questionnaire, and a prepaid return envelope. Non-respondents received one reminder 6–8 weeks later, containing the same material. Families who did not respond to either mailing were considered non-participants and excluded from further analysis. The study coordinator (RK) served as the designated contact person for questions, accessible via email.

### 2.3. Questionnaire

The questionnaire comprised several sections; this report focuses on the health-related quality of life data together with the accompanying medical and sociodemographic information. We first assessed medical information on patients’ cancer diagnoses and treatment received from the parents’ memories. Second, participants completed the Ukrainian version of the Pediatric Quality of Life Inventory (PedsQLTM) 4.0 Generic Core Scales, which included validated age-specific instruments (0–4 years, 5–7 years, 8–12 years, and 13–18 years). Last, we collected sociodemographic information on sex, age at the time of the questionnaire, and date of arrival in Switzerland. The questionnaire was developed in German and translated into Ukrainian by a certified translator (date translated 1 March 2024, UniTranslate, Switzerland, www.unitranslate.ch, last accessed 7 September 2026). Parents completed the survey on behalf of their younger children; for older children (age > 4 years), parents and patients completed it jointly.

### 2.4. Pediatric Quality of Life Inventory (PedsQLTM) 4.0 Generic Core Scales

PedsQLTM is a 23-item multidimensional assessment tool with four dimensions (1) physical functioning (8 items), (2) emotional functioning (5 items), (3) social functioning (5 items), and (4) school functioning (5 items). The scales are assessed in parallel via parent and child self-report. Child self-reports are structured into three age groups (5–7 years (young child), ages 8–12 years (child), and ages 13–18 years (adolescent). Parent reports are structured in 4 age groups (2–4 years (toddler), 5–7 years (young child), 8–12 years (child), and 13–18 years (adolescent)). The items for each of the forms are essentially identical but differ in language according to the developmental stage and grammar of the participant. The instructions ask the participant to assess if the items have been a problem during the last month on a 5-point Likert scale (0 never a problem; 1 almost never a problem; 2 sometimes a problem; 3 often a problem; 4 almost always a problem). In the young child age group the scale was reworded and simplified age-appropriately to a three-point scale (0 not at all a problem; 2 sometimes a problem; 4 a lot of a problem) and was complemented by visual aids (happy face, neutral face and sad face). For calculating the scale scores, items are reverse-scored and transformed to a 0–100 scale (0 = 100; 1 = 75; 2 = 50; 3 = 25; 4 = 0); thus, a higher score indicates better HRQoL. The psychosocial health summary score consists of the sum of the scores of items answered in the emotional, social, and school functioning scales; the physical health summary score contains the physical functioning dimension scale score; and the total score contains all four dimensions [13,14].

We compared the overall scale scores against published cutoff scores for the PedsQL™ 4.0 to aid interpretation [15]. For major chronic conditions, including but not limited to cancer, Huang et al. recommend a cutoff of 77 for children < 8 years and 70 for children ≥ 8 years for the overall scale score; scores below these cutoffs indicate potentially clinically relevant impairment [15]. The cutoffs were derived in a US population using the parent proxy-report version. We therefore used them descriptively rather than as diagnostic thresholds, primarily to compare our parent-reported scores and, for orientation, the self-reported scores.

### 2.5. Medical Data Collection

Clinical data (diagnosis, age at diagnosis, and treatment received) were abstracted from medical records at each participating center. Data were collected by the study investigators during site visits, with information extracted from either electronic or paper records, depending on local systems.

### 2.6. Statistical Analysis

Descriptive statistics were used throughout this exploratory analysis. We used Chi-square test to compare participants’ characteristics by responder status. We summarized HRQoL as mean (SD) stratified by age group, as assessed by the PedsQL^TM^ for physical, psychosocial, and overall health via parent report and self-report. Differences between age groups were explored using one-way ANOVA; given the small subgroup sizes (*N* = 2–6), these comparisons are considered exploratory and are interpreted with caution. We used Stata (version 15, Stata Corporation, Austin, TX, USA) for all analyses.

## 3. Results

A total of 23 eligible participants across five Swiss pediatric Oncology Group hospitals were contacted. Fourteen families (61%) responded, while nine (39%) did not. Among non-respondents, one family had returned to Ukraine, one address was incorrect, and seven did not reply (Supplementary Figure S1).

Participants’ characteristics are summarized in Table 1. The mean age at study participation was 12.0 (SD 5.5) years for respondents and 13.3 (SD 4.5) years for non-respondents (*p* = 0.567). The mean time from arrival in Switzerland to questionnaire completion was 21.7 months (SD 9.9). Sex distribution differed significantly, with a higher proportion of males among participants (64% vs. 11%; *p* = 0.031). Cancer diagnoses varied: leukemia/lymphoma was more frequent in participants (71% vs. 11%), while solid and CNS tumors were more common in non-respondents (44% vs. 21% and 33% vs. 8% in non-respondents vs. participants, respectively; *p* = 0.032). The mean age at cancer diagnosis did not differ between groups (9.6 vs. 9.4 years, *p* = 0.908). Treatment modalities showed non-significant differences.

Health-related quality of life (HRQoL) by age group is presented in Table 2 and Table 3. Parent-reported PedsQL™ scores demonstrated a trend toward lower physical health with increasing age. Toddlers (2–4 years) scored highest in physical functioning (mean 98, SD 2.2), while adolescents (13–18 years) scored the lowest (mean 64, SD 31.4). A similar age-related decline was observed in overall health (88 vs. 68). Psychosocial scores showed less variation, ranging from 83 (toddlers) to 70 (adolescents). This decline could only be observed in the physical scale score when young children, children and adolescents made self-reports, psychosocial scale scores and overall scale scores were similar in all age groups when self-reported. Parent–child agreement on HRQoL showed almost no discrepancies between reports in young children, children and adolescents. Overall, self-reported scores were similar to parent proxy-reports. For example, mean overall health was rated at 73 (SD 21.6) by parents compared to 72 (SD 39.1) by the children and adolescents themselves (Table 2 and Table 3).

Compared to published cutoffs for major chronic conditions (77 for children < 8 years, 70 for children ≥ 8 years) [14], mean overall scale scores fell below the respective cutoffs in young children aged 5–7 years (parent report 75, self-report 72) and in adolescents aged 13–18 years (parent report 68, self-report 67), whereas mean scores in toddlers aged 2–4 years (parent report 88) and children aged 8–12 years (parent report 74, self-report 78) remained above them. As these are group means rather than individual reports, and as the subgroups are very small (*N* = 2–6) with wide standard deviations, this comparison is descriptive only.

## 4. Discussion

This is the first exploratory study reporting on HRQoL in Ukrainian child and adolescent cancer patients relocated to other countries, specifically to Switzerland, after the start of the Ukrainian–Russian war in 2022. HRQoL scores suggest an age-related decline, particularly in physical functioning, while psychosocial functioning remained relatively stable but tended to be lower among adolescents. Parents rated their child’s HRQoL similarly to self-reports. Given the exploratory design and the small, displaced cohort, these patterns are descriptive and cannot be attributed to any single cause.

From earlier studies we know that children and adolescents with cancer and their families might have impaired quality of life [7,8], especially compared to healthy subjects. In our study we also found that reported HRQoL according to the PedsQL^TM^ overall self-reported scores were lower than in healthy Ukrainian children (Supplementary Table S1) [14] and similar to other pediatric cancer populations [13]. In our study, parents rated their child’s HRQoL similarly to self-reports, which suggests that parents have a good understanding of their child’s HRQoL, even in such combined stressful situations. In other studies, parents tended to rate their healthy child’s HRQoL lower than the children’s or adolescents’ self-reported scores [14]. This difference could represent a closer parental involvement during treatment in a foreign country. Self-reported HRQoL scores showed an age-related decline in our study population, especially for physical functioning and the overall scale score, while psychosocial functioning remained relatively stable but tended to be lower among adolescents. This observation could come from increasing awareness of physical limitations while aging but also from different perceptions of the changes happening around the move away from home. Adolescents might also have higher expectations for physical performance than younger children. The lower psychosocial scale score among adolescents might reflect increasing psychosocial stressors such as changes in location and peer environment from the home country to the setting in Switzerland, or unclear educational, familial, and career development in the future. Future studies could investigate longitudinal trends in HRQoL to see if the perception of physical health improves in UCC or other migrating child and adolescent cancer patient populations over time.

Notably, in children aged 5–7 years and adolescents aged 13–18 years, mean overall scale scores fell below published clinical cutoffs for major chronic conditions on both parent- and self-report; the 8–12 year group scored above the cutoff. Although based on very small subgroups, this consistency across reporters raises the possibility that these two age groups may be worth assessing more closely in clinical practice. Further, it will be important to research the factors influencing lower HRQoL scores among adolescents; for this, a larger study population will be needed.

Several limitations should be considered. First, the sample size is small; this reflects the finite nature of the population, as the study included all eligible UCC under active treatment at five of the nine Swiss pediatric oncology centers. Further stratification or regression analysis to identify specific cancer- or cancer-treatment-related factors contributing to lower HRQoL or higher health care needs was thus not possible, and the results should be regarded as exploratory and hypothesis-generating. Second, the study lacks a matched control group, as recruiting non-displaced Ukrainian children and adolescents with cancer or a matched Swiss pediatric oncology cohort was not feasible in this project and would have raised considerable practical and ethical challenges. We compared the observed scores with a published, validated cohort of healthy Ukrainian children and with previously reported pediatric cancer populations; however, these are external references rather than matched controls. Consequently, the observed HRQoL patterns cannot be attributed specifically to displacement, and the respective contributions of displacement, cancer and its treatment, developmental age, and other factors cannot be disentangled. Strengths of this study include its multicenter cohort design in Switzerland, including all UCC of five pediatric oncology centers (Aarau, Basel, Lausanne, Lucerne, St. Gallen) representing all major regions of Switzerland. Further, the study reached a relatively high response rate among contacted UCC, which is similar to or even slightly higher than other questionnaire-based cohort studies in Switzerland [16,17].

Reaching this population was feasible largely because of how pediatric oncology is organized in Switzerland. Childhood cancer patients are normally treated at a Swiss Pediatric Oncology Group center, so UCC entered routine hospital records on arrival at one of the participating clinics and could be contacted through their treating center rather than through community or refugee networks. As all were under active treatment, they remained in regular contact with that center. Certified translation of all study materials and a single-named study coordinator were, in our experience, essential. Displacement nevertheless limits reach: one address was no longer valid, and one family had returned to Ukraine, illustrating that a proportion of families become unreachable within months even in a well-organized setting.

## 5. Conclusions

In this exploratory Brief Report, HRQoL among UCC living in Switzerland declined with age, most clearly in physical functioning, while psychosocial functioning was more stable but tended to be lower among adolescents. Parents rated their child’s HRQoL similarly to self-reports. These exploratory findings suggest several practical considerations for centers receiving displaced patients in future migration crises. HRQoL should be assessed routinely with short, validated, age-specific instruments in the family’s own language, rather than being assumed. Where self-report is not feasible, parent proxy-reports appear to offer a usable alternative, and harmonized documentation across centers would facilitate future research. Supportive care should be age-tailored, and attention to physical rehabilitation and to psychological stressors related to displacement may be relevant. Larger, controlled, and longitudinal studies are needed to confirm these observations and to identify factors associated with lower HRQoL in displaced children and adolescents with cancer.

## Figures and Tables

**Table 1:** Characteristics of participants and non-participants.

*N* = 23	Participants		Non-Participants		
	*N* = 14	%	*N* = 9	%	*p*-Value ^+^
**Sex** Male Female	9 5	64% 36%	1 8	11% 89%	0.031
**Mean age at study** **in years (SD)**	12.0	(5.5)	13.3	(4.5)	0.567
**Mean time from arrival in Switzerland to questionnaire completion** **in months (SD)** **‡**	21.7	(9.9)			
**Cancer Diagnosis**					
Leukemia/Lymphoma	10	71%	1	11%	0.032
Solid Tumors	3	21%	4	44%	
CNS Tumors	1	8%	3	33%	
No available data			1	11%	
**Mean age at time of cancer diagnosis in years (SD)**	9.6	(5.8)	9.4	(3.6)	0.908
Treatment * Chemotherapy Radiotherapy HSCT Tumor surgery	13 2 1 3	93% 14% 7% 21%	6 0 1 5	67% 0% 11% 56%	0.227 0.241 0.407 0.073

* Combined treatments were possible. ^+^ derived from Chi-square tests. ‡ Date of arrival in Switzerland was self-reported in the questionnaire and is therefore available for participants only; two participants did not provide this information (*N* = 12). Abbreviations: CNS, central nervous system; HSCT, hematopoietic stem cell transplantation (includes auto- and allo-transplantations); *N*, number; SD, standard deviation.

**Table 2:** PedsQL^TM^ 4.0 Generic Core Scales for physical, psychosocial and overall health by parent report stratified by age group, displayed by mean (SD).

*N* = 14	Toddlers (Ages 2–4) *N* = 2		Young Children (Ages 5–7) *N* = 2		Children (Ages 8–12) *N* = 4		Adolescents (Ages 13–18) *N* = 6		*p*-Value *
	Mean	(SD)	Mean	(SD)	Mean	(SD)	Mean	(SD)	
Psychosocial Scale Score (0–100)	83	(12.9)	71	(34.5)	73	(19.8)	70	(23)	0.919
Physical Scale Score (0–100)	98	(2.2)	81	(26.5)	76	(18.1)	64	(31.4)	0.452
Overall Scale Score (0–100)	88	(7.7)	75	(31.4)	74	(19.1)	68	(25.6)	0.767

* *p*-values were generated using one-way ANOVA comparing differences between age groups; given the small subgroup sizes (*N* = 2–6), these *p*-values are exploratory only. Abbreviations: *N*, number; SD, standard deviation.

**Table 3:** PedsQL^TM^ 4.0 Generic Core Scales for physical, psychosocial and overall health by parent report compared to self-report, displayed by mean (SD).

	Parent Report		Self Report								
	Overall *N* = 14		Overall *N* = 12		Young Children (Ages 5–7) *N* = 2		Children (Ages 8–12) *N* = 4		Adolescents (Ages 13–18) *N* = 6		*p*-Value *
	Mean	(SD)	Mean	(SD)	Mean	(SD)	Mean	(SD)	Mean	(SD)	
Psychosocial Scale Score (0–100)	73	(20.5)	73	(23.4)	68	(38.8)	81	(22.0)	69	(23.2)	0.744
Physical Scale Score (0–100)	75	(25.6)	71	(26)	81	(26.5)	76	(18.1)	64	(31.4)	0.681
Overall Scale Score (0–100)	73	(21.6)	72	(39.1)	72	(34.6)	78	(19.7)	67	(25.6)	0.801

* *p*-values were generated using one-way ANOVA comparing differences between age groups; given the small subgroup sizes (*N* = 2–6), these *p*-values are exploratory only. Abbreviations: *N* number; SD standard deviation.

## Data Availability

The dataset presented in this article is not readily available because of privacy reasons.

## Supplementary Materials

The following supporting information can be downloaded at: https://www.mdpi.com/article/10.3390/curroncol33090553/s1. Figure S1: Flow Chart of mailing to participants; Table S1: PedsQLTM 4.0 Generic Core Scales for physical, psychosocial and overall health by parent report and self-report compared to a validated cohort of healthy Ukrainian subjects.

## Abbreviations

The following abbreviations are used in this manuscript:

EKOS	Ethics Committee of eastern Switzerland
HRQoL	Health-related quality of life
ICCC-3	International Classification of Childhood Cancer, Third Edition
NGO	Non-governmental organization
PedsQLTM	Pediatric Quality of Life Inventory 4.0 Generic Core Scales
SPOG	Swiss Pediatric Oncology Group
SSPHO	Swiss Society of Pediatric Hematology and Oncology
UCC	Ukrainian children and adolescents with cancer
