# Supplementary material for: Quality of Life in Ukrainian Children and Adolescents with Cancer Relocated to Switzerland During the Russian–Ukrainian War: An Exploratory Multicenter Study
Source: Curr Oncol. 2026 Sep 11;33(9):553. doi: 10.3390/curroncol33090553 (PMC13605239; doi:10.3390/curroncol33090553)
Supplement: Supplementary file 1 [file curroncol-33-00553-s001.zip › curroncol-4497975-supplementary.pdf]

Supplementary Material

Quality of life in Ukrainian children and adolescents with cancer relocated to Switzerland during the Russian–Ukrainian war: an exploratory multicenter study

Rahel Kasteler, MD PhD [1,2] ; Dr. med. Ahmed Farrag [3,4]; Dr. med Andreas Klein-Franke [5]; Dr. med. Calogero Mazzara [6]; PD Dr. med. Francesco Ceppi [6]; Dr. med. Cornelia Vetter [1]; Prof. Dr. med. Nicolas von der Weid [7]; Prof. Dr. med. Katrin Scheinemann [1,8]

Affiliations

1. Pediatric Hematology-Oncology Center, Children's Hospital of Eastern Switzerland, St Gallen, Switzerland
2. Department of Oncology, Hematology, Immunology, Stem Cell Transplantation and Somatic Gene Therapy, University Children's Hospital Zurich-Eleonore Foundation, Zurich, Switzerland
3. Department of Pediatrics, Division of Pediatric Hematology and Oncology, Children's Hospital of Central Switzerland, Lucerne, Switzerland
4. Pediatric Oncology Department, South Egypt Cancer Institute, Assiut University, Assiut Egypt.
5. Division of Pediatric Hematology and Oncology, Cantonal Hospital Aarau, Aarau, Switzerland
6. Department Woman-Mother-Child, Pediatric Hematology-Oncology Unit, Division of Pediatrics Lausanne University Hospital (CHUV) and University of Lausanne (UNIL), Lausanne, Switzerland
7. Department of Pediatric Hematology and Oncology, University Children's Hospital Basel (UKBB) and University of Basel, Basel, Switzerland
8. Faculty of Health Sciences and Medicine, University of Lucerne, Lucerne, Switzerland

Supplementary Table S1 PedsQL™ 4.0 Generic Core Scales for physical, psychosocial and overall health by parent report and self-report compared to a validated cohort of healthy Ukrainian subjects.

	Ukrainian Children with Cancer treated in Switzerland				Healthy Ukrainian Children comparison[1]			
	Parent Report		Self Report Over all age groups		Parent Report		Self Report Over all age groups	
	N=14 mean	(SD)	N=12 mean	(SD)	N=41 Mean	(SD)	N=41 Mean	(SD)
Psychosocial Scale Score (0-100)	73	(20.5)	73	(23.4)	69.73	(14.71)	77.36	(21.7)
Physical Scale Score (0-100)	75	(25.6)	71	(26)	75.48	(17.15)	76.89	(17.40)
Overall Scale Score (0-100)	73	(21.6)	72	(39.1)	71.77	(13.76)	77.36	(21.73)

[1] Results from the following publication: Kovalchuk T. Validation of the Ukrainian version of the PedsQL™ 4.0 Generic Core Scales in children and adolescents with vasovagal syncope. Pediatria Polska - Polish Journal of Paediatrics, 2020. p. 112-20.

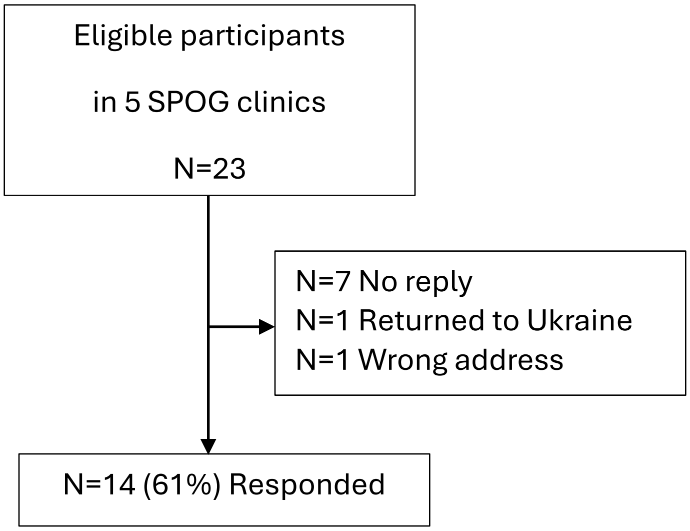

Supplementary Figure S1 Flow Chart of mailing to participants

Abbreviations: SPOG Swiss Pediatric Oncology Group
